# A Biologically-validated HCV E1E2 Heterodimer Structural Model

**DOI:** 10.1038/s41598-017-00320-7

**Published:** 2017-03-16

**Authors:** Matteo Castelli, Nicola Clementi, Jennifer Pfaff, Giuseppe A. Sautto, Roberta A. Diotti, Roberto Burioni, Benjamin J. Doranz, Matteo Dal Peraro, Massimo Clementi, Nicasio Mancini

**Affiliations:** 1 Laboratory of Microbiology and Virology, Università “Vita-Salute” San Raffaele, Via Olgettina 58, 20132 Milano, Italy; 2 Integral Molecular, 3711 Market St #900, Philadelphia, PA 19104 USA; 3 Laboratory for Biomolecular Modeling, Institute of Bioengineering, School of Life Sciences, Ecole Polytechnique Fédérale de Lausanne, Route Cantonale, 1015 Lausanne, Switzerland; 4 Swiss Institute of Bioinformatics, Lausanne, Switzerland

## Abstract

The design of vaccine strategies and the development of drugs targeting the early stages of Hepatitis C virus (HCV) infection are hampered by the lack of structural information about its surface glycoproteins E1 and E2, the two constituents of HCV entry machinery. Despite the recent crystal resolution of limited versions of both proteins in truncated form, a complete picture of the E1E2 complex is still missing. Here we combined deep computational analysis of E1E2 secondary, tertiary and quaternary structure with functional and immunological mutational analysis across E1E2 in order to propose an *in silico* model for the ectodomain of the E1E2 heterodimer. Our model describes E1-E2 ectodomain dimerization interfaces, provides a structural explanation of E1 and E2 immunogenicity and sheds light on the molecular processes and disulfide bridges isomerization underlying the conformational changes required for fusion. Comprehensive alanine mutational analysis across 553 residues of E1E2 also resulted in identifying the epitope maps of diverse mAbs and the disulfide connectivity underlying E1E2 native conformation. The predicted structure unveils E1 and E2 structures in complex, thus representing a step towards the rational design of immunogens and drugs inhibiting HCV entry.

## Introduction

Hepatitis C Virus (HCV) is an enveloped, positive-stranded RNA virus belonging to the *Hepacivirus* genus in the Flaviviridae family. It presents two highly glycosylated surface proteins, E1 and E2, the study of which has been impaired by the difficulties of both culturing HCV *in vitro* and obtaining E1 and E2 atomic structures.

Among the Flaviviridae family, the most well-characterized viruses belong to the *Flavivirus* genus (i.e. Dengue and Tick-borne Encephalitis Virus (TBEV)). The outer morphology hallmarks of this genus are a smooth surface and an icosahedral-like symmetry induced by the sequential assembly of envelope-associated glycoprotein E into ordered oligomers. *Flavivirus* glycoproteins E are prototypical class II fusion proteins, presenting an elongated, three-domain topology parallel to the envelope and a tail-to-head assembly in homodimers, the pre-fusion functional unit mediating virus entry. Furthermore, all *Flavivirus* have a second surface protein, prM, which prevents undesired fusion events during virion maturation and is subsequently cleaved by host proteases to achieve the mature conformation^1–3^.

Less well studied Flaviviridae viruses belonging to *Hepacivirus, Pestivirus* and *Pegivirus* genera have been long thought to share analogous structures and entry process to the *Flavivirus* genus. However, *Pestivirus* Bovine Viral Diarrhea Virus (BVDV) envelope glycoprotein E2 has been demonstrated to adopt a unique conformation, casting doubts on common surface structures among Flaviviridae^4^. Similarly, HCV/E2 has been long considered the primary mediator of virus entry, with E1 mostly acting as a molecular chaperone for E2. However, several recent papers have highlighted that none of the *Flavivirus* outer structure features and functions may apply to HCV. For example, Catanese *et al.* studied the external morphology of HCV particles produced by primary human hepatocytes and found them not to adopt an icosahedral-like symmetry in cryo-electron tomography (cryo-ET) reconstructions; they also have identified electron-dense, spike-like structures incompatible with a smooth icosahedral surface^5^. Furthermore, two recent crystallographic structures of E2 core (E2c) and of E1 N-terminal segment found no structural homology with class II fusion proteins^6–8^. Finally, Falson *et al*. have reported that a large fraction of E1 on the envelope of both cell culture-derived HCV (HCVcc) and pseudoparticles (HCVpp) is in a homotrimeric state, suggesting that the functional unit is formed by a trimer of covalently-bound E1E2 heterodimers, where an E1 trimer constitutes the heterohexamer core^9^. Together with the increasing evidence of E1 possessing a fusogenic region, these findings on E1 and E2 structures, oligomerization state and function highlight the absence of a reliable model for HCV entry machinery^10–14^. Furthermore, although the existing E1 and E2 crystal structures represent a significant improvement in the comprehension of functionally relevant E2 domains (i.e. the Ig-like domain that includes the CD81 binding site (CD81bs) and some neutralizing epitopes), they are not representative of the native, unmodified structure (i.e. they are truncated and expressed without the other partner protein E1 or E2) and cannot be completely validated through functional or immunological testing^15^.

We have previously reported the pattern of disulfide bridges defining E2 native folding through the analysis of the reactivity of E2 cysteine to alanine (C-to-A) mutants against a panel of human monoclonal antibodies (mAbs). Importantly, E2 mutants in these studies were co-expressed with E1 and the tested mAbs were isolated from a phage display human antibody library generated from a patient chronically infected with HCV 1b genotype. All of the mAbs used recognize epitopes present on phylogenetically distant HCV genotypes and are stratified into non-neutralizers (e8, e10), weak neutralizers (e509) and potent neutralizers (e20, e137, e301)^16–20^. H60, a murine conformational antibody able to bind E2 only when complexed with E1, was tested as well^21, 22^. Considering the origin of these mAbs, together with their recognition of conformational epitopes, they represent ideal probes to test the native conformation of E1E2. This data allowed us to propose the appropriate disulfide connectivity of native E2, resolving the discrepancies in E2c crystal structures^15^.

Here we report the reactivity features of our mAb panel against each of 553 X-to-A single mutant in the full-length (FL) E1E2 of H77 strain. The obtained data led to the identification of E1 and E1E2 disulfide connectivity and the fine epitope definition of our mAbs on E2. The E1E2 disulfide connectivity pattern, together with several protein secondary structure prediction tools and evolutionary coupling (EC) algorithms, was subsequently used to generate a model of the fully-glycosylated E1 and E2 ectodomains in their heterodimeric form. The structural model was validated through the mapping of biologically and immunologically relevant domains to guarantee a direct relation between the predicted structure and its functions.

The proposed E1E2 heterodimer structure provides insights into HCV entry machinery, paving the way for a more accurate characterization of the HCV entry process and, consequently, the development of prophylactic and therapeutic strategies that can inhibit early stages of HCV infection.

## Results

### E1 disulfide connectivity

**Figure 1:**
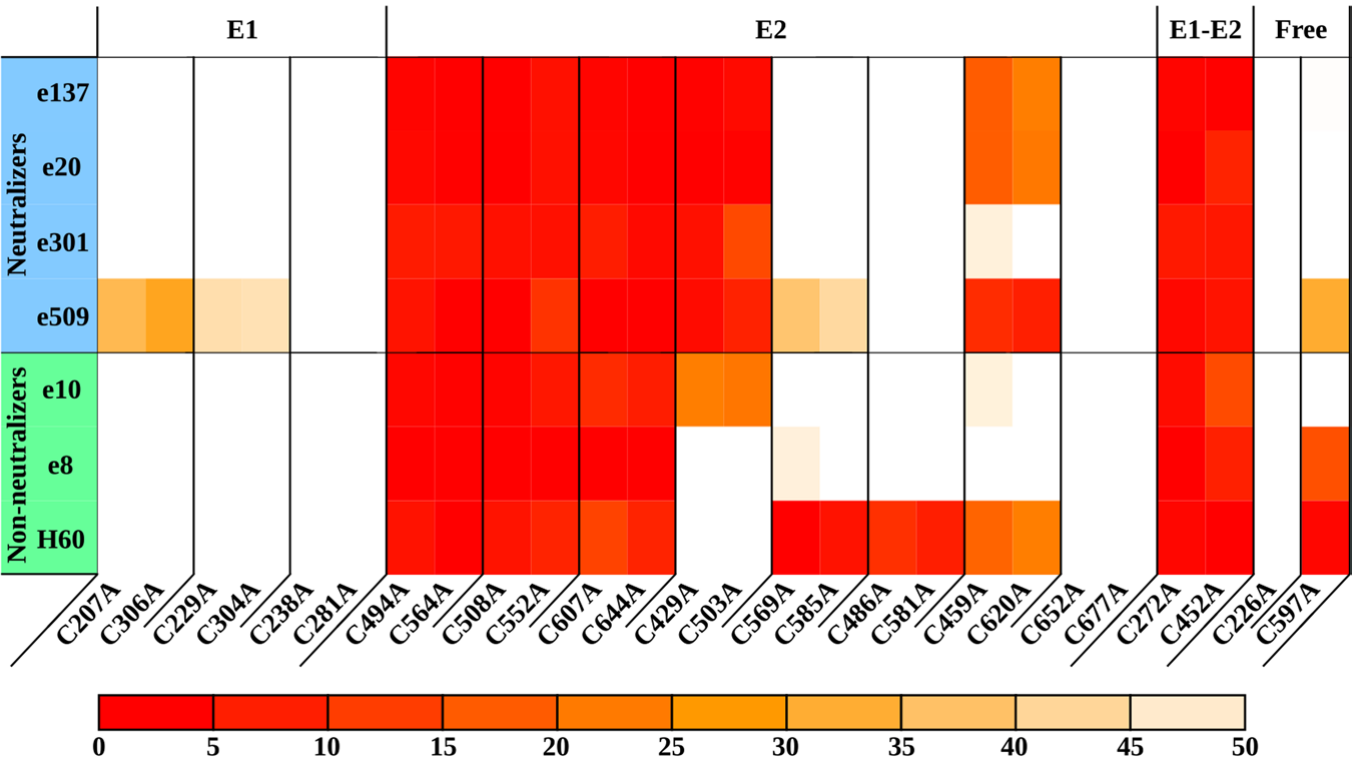
C-to-A reactivity pattern. Antibodies are divided into neutralizers and non-neutralizers. Each cysteine mutant was tested against the mAb panel. Disulfide bridges and free cysteines selected on the basis of their reactivity are reported. Reactivities below 50% compared to WT are depicted as a heatmap spanning from light orange (50%) to red (0%).

HCV E1 and E2 ectodomains contain eight and eighteen cysteines, respectively. The disulfide bridges involving both intra- and inter-molecular bonds are speculated to depend on the form (i.e. full length – FL, soluble, truncated) used for expression, the co-expression of E1 and E2 and the system used^7, 8, 23^. Moreover, the connectivity of the disulfide bonds is believed to change upon host receptor binding^24^. We have previously described the disulfide connectivity underlying E2 native conformation using a panel of mAbs against each C-to-A mutant in FL E2^15^. Here we report the effect of each E1 single-point cysteine mutant against the same panel, leading to the identification of E1 native connectivity and of an E1E2 inter-molecular bond necessary for the assembly of E1 and E2 into heterodimers (Fig. 1).

**Figure 2:**
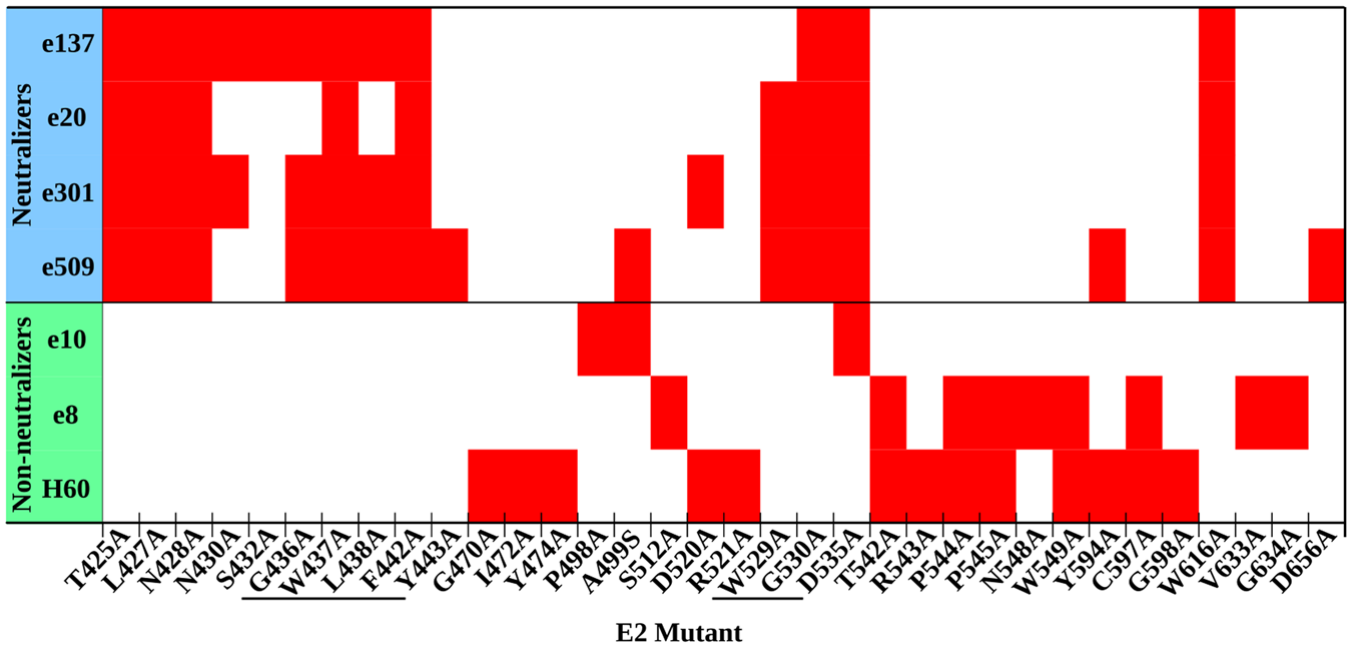
Critical residues abrogating mAb binding. Antibodies are divided into neutralizers and non-neutralizers. Epitope residues are marked in red, while residues that are part of the CD81bs are underlined. The threshold below which a decrease in reactivity defines a residue as part of an epitope was determined for each antibody (e137 < 15%, e20 < 20%, e301 < 20%, e509 < 15%, e10 < 30%, e8 < 20%, H60 < 15%).

Cysteines involved in a disulfide bridge were determined evaluating in immunofluorescence the reactivity pattern of each C-to-A mutant against the mAb panel compared to the wild-type (WT). Interestingly, only C597 was identified as part of an epitope (Fig. 2); therefore, the reactivity pattern of a mutated cysteine is ascribable to the disruption of a disulfide bridge rather than to a direct effect on a mAb binding. When more than two C-to-A mutants showed analogous patterns, two different strategies were applied: E2 disulfide connectivity assignment was aided by E2c, while for E1 the correct pattern was driven by the predicted tertiary structure^15^.

Although the reactivity profile could not readily provide unambiguous information for all E1 cysteines, it suggested the presence of an inter-molecular disulfide bond involving residues E1 C272 and E2 C452. Specifically, the previously reported E2 reactivity pattern highlighted how the C452A mutation abrogated the binding of all the tested conformation-sensitive mAbs and remained uncoupled in E2. The same binding properties were detected for E1 C272A mutant, strongly suggesting the formation of a C272-C452 disulfide bridge involved in the functionally-competent E1E2 assembly. All other C-to-A variants led to either a reactivity pattern comparable to the wild-type (WT) or the marked reduction of mAb e509 binding. However, as described later, the combination of reactivity data with the prediction of E1 secondary and tertiary structure led to the identification of three disulfide bonds, C207-C306, C229-C304 and C238-C281. Furthermore, C226 does not have a partner and thus it can be speculated that it remains in its free state; interestingly, the presence of at least one free cysteine is fundamental in driving the conformational changes the HCV entry machinery undergoes upon binding to CD81 during virus fusion^24^. Altogether, the reactivity of E1 and E2 C-to-A mutants allows a more accurate depiction of the disulfide connectivity that keeps the E1E2 dimer in its native conformation, providing a starting point to predict the complex structure.

### Definition of mAb epitopes

Cysteine to alanine mutants are part of a larger alanine scanning analysis that we conducted covering the entire E1 and E2 proteins. Each of 553 X-to-A mutants was tested against seven antibodies, leading to the fine identification of the epitope residues for each mAb. All the mAbs included in the panel have been thoroughly characterized before in terms of neutralizing activity and genotype specificity, leading to their classification as non-neutralizers (e8, e10), weak neutralizer (e509) or potent neutralizers (e20, e301 and e137). All mAbs recognize HCV E1E2 belonging to a variety of virus genotypes, indicating a high conservation at their binding site on E2^16–20^.

The identification of E2 residues belonging to a mAb epitope relied on the binding reduction induced by each X-to-A mutant compared to the WT. The threshold below which a residue is considered as part of the epitope was individually selected for each antibody based on an established decision tree designed to yield a biophysically appropriate number of critical residues (3–13) that are the major energetic contributors to the antibody/antigen interaction^25^. This approach has been used for several conformational mAbs specific for viral surface proteins and validated through X-ray crystallography and cryo-electron microscopy studies^26–30^. Epitope residues for each mAb are depicted in Fig. 2; as highlighted, all neutralizing antibodies described here act by interfering with E2-CD81 interaction through the recognition of residues included in the so-called epitope II and epitope III^31–33^. Importantly, all the neutralizing mAbs recognize the majority of the highly conserved residues belonging to the CD81bs; of the designated fundamental residues for CD81 binding (420, 436–443, 527, 529, 530 and 535), only W420 and Y527 are indeed not directly recognized by our mAbs. The role of these residues in HCV entry, together with their conservation among different genotypes, provides a molecular explanation for the potent and broad neutralizing activity of mAbs e20, e137 and e301.

The epitopes of three non-neutralizing mAbs, e8, e10 and H60, were characterized as well. The recognized residues are all located outside the functionally relevant E2 domains, with e8 and H60 having partially overlapping epitopes, while that of e10 is distinct. In details, they all recognize residues located on the opposite side of the CD81bs in the Ig-like domain, with e10 epitope being centered around residues 498, 499, 520, 521 and 535, while e8 and H60 both bind residues located in the 542–549 region. In addition, the e8 epitope includes residues in the C-terminal E2c β-sheet (residues 633 and 634). Most notably, other H60 epitope residues lie in HVR2 (470, 472 and 474) and near the IgVR (594, 597 and 598). Since H60 is able to bind to E2 only when it is complexed with E1, it can be speculated that HVR2 and IgVR, whose structures remain unsolved, are in close proximity only when the native E1E2 quaternary structure is formed^21^. Given the role of HVR2 and IgVR in E1E2 heterodimerization, H60 represents the ideal probe for E1E2 heterodimer native folding^34^.

### E1E2 secondary structure analysis

**Figure 3:**
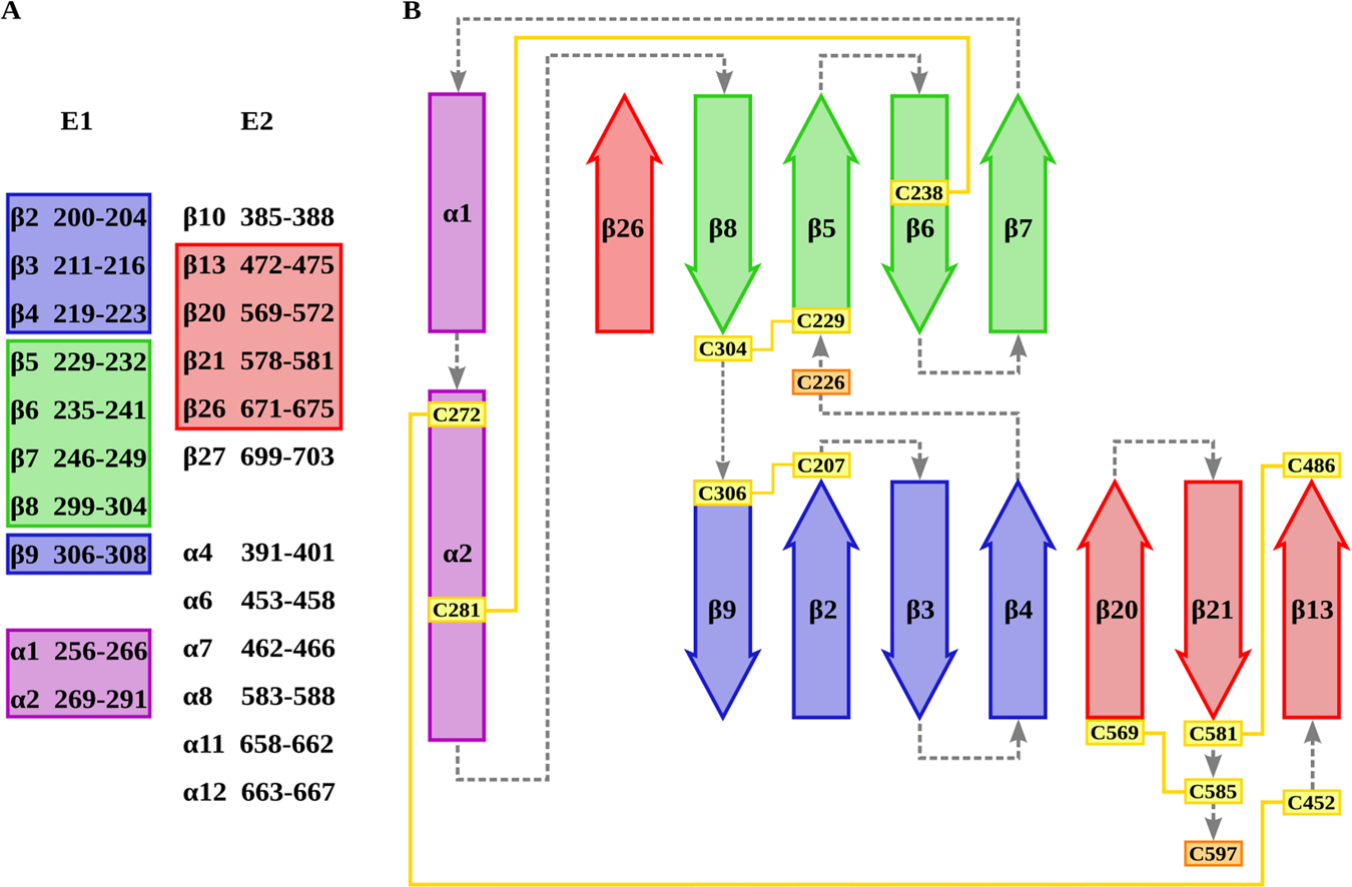
Secondary and tertiary structure prediction. In (**A**) all newly identified secondary structures in E1 and E2 are reported. **(B**) Secondary, tertiary and quaternary arrangement of E1 and E1E2 interfaces. In both panels, previously characterized domains are omitted. Strands and helices, respectively represented as arrows and rectangles, follow the same color scheme in both panels. Successive secondary structure elements are connected by dashed gray lines. Cysteines involved in disulfide bonds are boxed in yellow and connected by yellow lines, free cysteines are boxed in orange.

The final goal of this study is to model the structure of the heterodimer formed by E1 and E2 ectodomains, spanning E1 residues 192–327 and E2 384–711. In order to first predict E1 and E2 secondary structure, we applied several algorithms to FL E1E2 multiple sequence alignment (MSA) when possible and to H77 E1E2 sequence for algorithms taking a single sequence as input. The predictions allowed us to identify a conserved secondary structure pattern; however, some small domains were ambiguously identified and a majority voting approach had to be used to assure prediction quality for those regions. The final secondary structure used for further studies is depicted in Fig. 3 and Figure S1. Compared to E2c crystal structure, secondary structures were correctly identified after merging all the predictions, except for β-strands 636–644; here, and for secondary structure boundaries in general, our final prediction followed the secondary structure assignment from E2c crystal structure (PDB ID: 4MWF, 4WEB)^7, 8^. Also, the correct prediction for epitope I β-hairpin (PDB ID: 4GAG) and epitope II α-helix (PDB ID: 4HZL) strengthen our predictions^35, 36^.

Recently, the E1 N-terminal domain (residues 192–272, PDB ID: 4UOI) structure was determined; however, the construct was not validated either functionally or immunologically^6^. Moreover, it forms homodimers in solution, while FL E1 on virions and the entire ectodomain in solution were recently shown to homotrimerize^9^. Finally, none of the disulfide bonds highlighted in the E1 N-terminal structure matched our mAb reactivity pattern. Consequently, 4UOI secondary structure assignment was considered only when it matched with the predictions.

E2 ectodomain secondary structure analysis of previously uncharacterized domains highlighted several structured segments. Specifically, residues 385–388, 472–475, 569–572 and 578–581 in the hypervariable regions and 671–675 and 699–703 in the domain between E2c C-terminal and the membrane proximal domain (MPD) are predicted to be β-strands, while residues 393–402, 453–458, 462–466, 583–588, 658–662, 664–668, 684–697 and 706–710 adopt a helical conformation.

The same prediction revealed E1 ectodomain content in α-helices surrounded by short β-strands; helices span residues 256–266, 269–291 and 317–324, while β-strands are located in regions 193–196, 200–204, 211–216, 219–223, 229–232, 235–241, 246–249, 299–304 and 306–309.

### E1E2 evolutionary coupling analysis

In the past few years, several algorithms able to predict proximal residue couples in a protein tertiary structure starting from large protein MSA have been developed. Since interacting residues are predicted as a function of each pair's coevolutionary rate, prediction quality is greatly affected by protein length, the number of aligned sequences and sequence diversity^37^. Considering these parameters, FL E1E2 is a challenging target because of its length and the non-optimal sequence variability within the MSA.

Because of the limitations of each, we used several predictors simultaneously and selected the one that performed the best when compared with the E2c Ig-like domain contact map, calculated with a Cα-Cα distance threshold of 10 Å on the 4MWF structure. The comparison allowed at the same time to estimate the specific predictive power of each algorithm against E2c, providing a high degree of confidence for the prediction of those domains of unknown structure. The contact propensity map predictor embedded in the RaptorX server outperformed all other algorithms in terms of positive predictive value (PPV) considering either the full prediction or the long-range (Δ_ij_ > 24) contacts only (Figure S2), and was therefore selected to guide E1E2 tertiary structure prediction. It should be noted that the tested predictors performance is in agreement with the published benchmarking results and that RaptorX may have outperformed all other algorithms due to its multi-family EC analysis that allows high-quality prediction even for MSAs with low numerosity and/or diversity^38^.

### E1E2 β-pairing prediction

The E1 and E2 structures solved to date have used truncated and modified constructs expressed in the absence of the partner protein. Besides small peptides co-crystallized with specific antibodies, reliable structures are available only for 72 residues of the E2 Ig-like domain (residues 495–566) and 44 residues of the β-sheet comprising residues 601–644. Direct information on E1 and E2 full-length ectodomains and their structure when complexed in heterodimers is completely missing.

With the goal of predicting E1E2 ectodomain tertiary and quaternary structures, we first sought to determine β-strands pairing for all the unsolved domains by applying the recently developed bbcontacts algorithm^39^. At first, the algorithm was fed with E1E2 secondary structure and RaptorX predicted contact map to test its predictive power; bbcontacts succeeded in correctly identifying five out of the six E2c β-strand pairings that were defined by X-ray crystallography. Subsequently, the predicted contact map was manually modified to favor the formation of E2c β-strands correct pairing improving bbcontacts analysis for the entire E1E2 structure. In Fig. 3 are depicted the newly identified secondary and tertiary structures for E1E2; E2c known structures are all correctly predicted based on the solved crystal structure (data not shown). Of greater interest, the algorithm *de novo* predicted several short β-sheets in both E1 and E2 and highlighted the presence of two β-pairings involving strands from both E1 and E2.

Newly identified E2 β-strands lie in the hypervariable regions and close to the MPD. Specifically, HVR1 385–388 β-strand couples parallel to residues 413–417, while IgVR residues 569–572 and 578–581 form a β-hairpin, with the latter interacting antiparallel to HVR2 472–475 β-strand. Finally, the C-terminal β-strand 699–703 couples antiparallel to E2c 624–634.

E1 is predicted to form two β-sheets that surround the central 256–266 and 269–291 helices, each comprising four strands. One β-sheet is composed by residues 229–232, 235–241, 246–249 and 299–304, the other by residues 200–204, 211–216, 219–223 and 306–309. From our analysis, the predicted N-terminal strand 193–196 remains uncoupled, thus suggesting its erroneous secondary structure assignment; consequently, this amino acid stretch was represented as unstructured in our model.

The algorithm bbcontacts further identified two, inter-monomer, β-coupling involving strands 219–223 and 569–573 that lie antiparallel, while strands 299–303 and 671–675 are parallel.

The entire E1E2 predicted topology and the accuracy of secondary and tertiary structure assignment is reported in Figure S3.

The predicted β-pairing allowed us to also disentangle E1 disulfide connectivity for those cysteines with a non-unique reactivity pattern. Residues C207, C229, C304 and C306 all had the same reactivity pattern and could not be readily coupled. Bbcontacts predicted a four-stranded, antiparallel β-sheet involving 229–232, 235–241, 246–249 and 299–304; consequently, C229, C238 and C304 lay one next to the other and, since C229 and C304 have the same reactivity pattern, it can be speculated they are involved in a disulfide bond. Consequently, C207 is coupled to C306 and C238 to C281 (Fig. 1).

Altogether, information obtained through the bbcontacts algorithm led to the identification of E1E2 heterodimer tertiary and quaternary structure arrangements through the prediction of intra- and inter-monomer β-sheet pairing and allowed us to univocally couple E1 cysteines residues that were previously unassigned. Merging E1E2 native disulfide connectivity and secondary and tertiary structure prediction together with RaptorX top scoring residue couples provides a sufficient number of structural restraints to generate an HCV E1E2 heterodimer structural model.

### E1E2 heterodimer reconstruction and validation

So far, we have described the generation of secondary, tertiary and quaternary restraints at the residue level. However, to correctly model the structure using CNS software, which was similarly used by Marks *et al.* for protein modeling based on evolutionary coupling data, detailed restraints at the atomic level are required^37, 40, 41^. Secondary structures were forced applying both angle and distance restraints to the backbone atoms and forcing β-sheet hydrogen bonds formation applying both N-O and H-O restraints. Furthermore, tertiary and quaternary structures were restrained using Cβ-Cβ distances, either measured for E2c known structures or predicted by RaptorX.

The CNS software was fed with the linear, disulfide-linked, all-atom structures of E1 and E2 ectodomain monomers and with interatomic distance restraints. Most notably, we have included both N- and O-glycosylations to assure the native conformation. The glycan types and sites were selected based on the mass spectrometry analysis performed by Bräutigam *et al.* and Iacob *et al.* and, neglecting microheterogeneity for some glycosylation sites, N-glycosylations and O-glycosylations were represented as Man6 and (Hex)_1_(HexNAc)_1_(NeuAC)_2_, respectively^42, 43^.

Before performing the final structure simulation, RaptorX predicted couples were filtered to remove false positives; specifically, a first structure prediction was performed using all the aforementioned restraints together with the L/5 (93 residue couples), top-scoring couples. Those restraints that could not be minimized by CNS were removed; after two sequential simulation and filtering steps, all the remaining distances (43 couples) were correctly minimized.

Furthermore, three different systems were run to resolve the disulfide connectivity involving E2 residues C486, C569, C581 and C585, as they all abrogate H60 binding when mutated to alanine. Of the three systems, only the one carrying the C486-C581 and C569-C585 disulfide bridges led to a correct geometry and was used for the final model (Figure S4).

**Figure 4:**
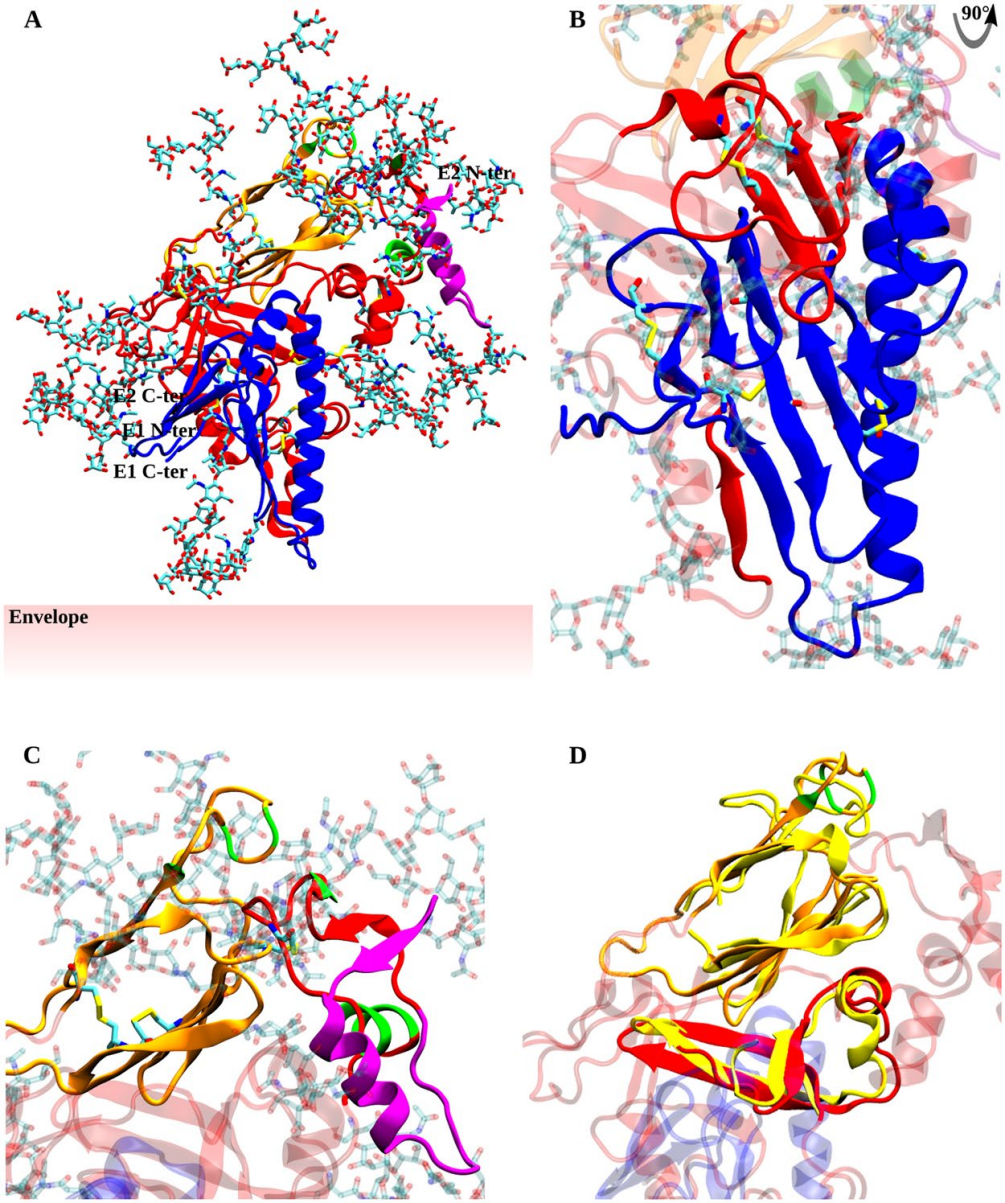
E1E2 heterodimer model. E1 and E2 structures are represented in cartoon and colored in blue and red, respectively. E2 HVR1, Ig-like domain and CD81bs are colored in purple, orange and green, respectively. Glycosylations are in licorice representation. (**A**) Overview of the entire, fully-glycosylated structure. (**B**) Details of E1, E1-E2 interfaces and the protective role of the variable regions HVR2 and IgVR on E1. These domains are depicted in solid colors, while the remaining E2 segments are transparent. (**C**) Protective role of HVR1 on CD81bs. Residues included in the CD81bs, Ig-like domain and HVR1 are in solid colors, all other regions are transparent. (**D**) Structural alignment of the E2c structured portions and the E1E2 model. 4MWF structure is aligned to the same regions of the model and colored in solid yellow. Model domains not superimposed to 4MWF are transparent. Glycosylations were removed for clarity.

The final restraints set was used to generate one hundred structures with the same parameters to explore the conformational space within the defined restraints, and these structures were subsequently clustered. The most populated cluster centroid was considered as the reference model and underwent a final unrestrained minimization step. The result is depicted in Fig. 4.

The model presented here reveals a compact structure for the E1E2 heterodimer, with the E2 Ig-like domain located apically, compatible with its role in the binding to CD81, and the HVR1 covering both the CD81bs and part of E1. The apical domains are highly glycosylated, while distal domains are more exposed, which is in agreement with their different exposure to the immune system (reviewed in ref. 44). E1 forms a compact structure with three distinct domain, a central, bipartite α-helix and two antiparallel, four-stranded β-sheets, that lie parallel to each other. Interestingly, E1 glycosylations partially shield both E1 itself and E2 distal regions.

**Figure 5:**
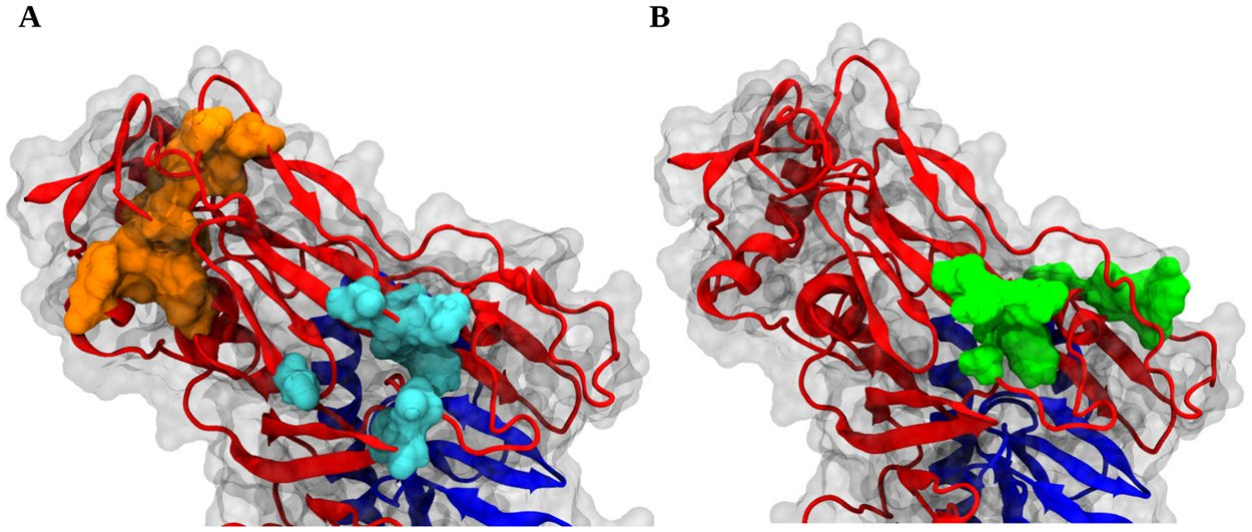
Epitope mapping on E1E2 model. E1 and E2 are represented in transparent surface and blue and red cartoon, respectively. **(A)** A non-neutralizing mAb (e8) and a broad neutralizing mAb (e137) epitopes are highlighted in cyan and orange, respectively. (**B**) H60 epitope is depicted in green.

To validate the model we mapped functional and immunological regions onto the structure, that are highlighted in Figs 4 and 5. All epitope residues are correctly exposed and clustered. Noticeably, the epitope of e8, a non-neutralizing mAb, localizes outside the functional regions and its center of mass is approximately 30 Å away from the epitope of e137, a broad neutralizer, providing a structural representation of the lack of neutralizing activity for e8 and the absence of reciprocal binding interference between the two mAbs. Furthermore, H60 epitope, which becomes available for binding only when E2 is complexed with E1, is well clustered and exposed (epitope mapping for the other tested mAbs is reported in Figure S5).

Overall, the E1E2 heterodimer model respects all the structural constraints previously determined by both *in vitro* and *in silico* techniques and, more importantly, all the biologically relevant regions can be coherently mapped on the model.

## Discussion

During recent years, the standard-of-care for HCV patients significantly improved in transitioning from the administration of non-specific drugs (PEG-interferon and ribavirin) with limited efficacy to the usage of highly selective inhibitors of HCV replicative complex and protease (reviewed in ref. 45). The development of specific inhibitors took advantage of the detailed structural characterization of HCV internal proteins. Conversely, the structure and oligomerization of the HCV surface proteins, and the functional steps leading to virus entry, remain largely uncharacterized. Consequently, HCV entry is not targeted by drugs in clinical use and vaccines against HCV have not been developed yet.

The limited structural information available for E1 and E2 is in large part a result of their intrinsic characteristics, being highly flexible and glycosylated, with a quaternary structure relying on a complex network of disulfide bridges and varying depending on the form and system used for *in vitro* expression. At the same time, the increasing evidence that HCV E1 and E2 does not adhere to the topology of existing Flavivirus structures hampers the possibility of inferring E1E2 structure by homology. In fact, recent data on E1E2 having a hexameric, spike-like conformation is intriguing, and suggests a topology that may actually be closer to the prototypical class I fusion proteins^9^.

In this paper, we have described a comprehensive model of the fully glycosylated E1E2 ectodomain in its heterodimeric, covalently-bound form, the functional unit on HCV virions at the basis of the formation of the heterohexamer proposed by Falson *et al*. The model takes advantage of the indirect structural information available in the literature, as well as of immunological data that we generated using a panel of conformational mAbs and state-of-the-art algorithms for protein structure prediction and modeling.

The model presents a compact topology characterized by a high degree of unstructured regions; E1 content in β-strands and α-helices is 25% and 27%, respectively, while for E2 is 26% and 10%. The secondary structure discrepancies between our model and circular dichroism experiments on E2 truncated forms are likely due to the usage of different constructs expressed in absence of E1; these aspects may have affected the local conformation of E2 and, therefore, its secondary structure content^23, 46^. As a first validation of the model, E2c Ig-like and C-terminal β-sheet structures are well maintained, retain their reciprocal orientation and locate apically in the heterodimer, as expected considering the role of E2c in binding to CD81.

Furthermore, our analyses and modeling strategy enables the proposal of the structure of those E1 and E2 domains with previously unknown structure. E2 HVR1 is highly flexible and exposed, as previously demonstrated by limited proteolysis and deuterium exchange experiments; however, two defined secondary structure elements were identified, an α-helix spanning residues 393–402 and a short 385–388 β-strand, that couples parallel to 413–417, which is part of epitope I β-hairpin^7^. These structured elements confer to HVR1 a compact, although flexible, conformation. Moreover, its apical localization is compatible with its dual role in both protecting the CD81bs from the humoral immune response and mediating early entry stages through the interaction with SR-BI, and allows for the first time to speculate that HVR1 has a direct role in protecting E1 as well. Interestingly, the predicted β-sheet involving epitope I suggests that its conformation in native conditions is a β-hairpin and, consequently, that the partially or completely unfolded conformations highlighted in co-crystallization experiments are either induced by the binding to different mAbs or functionally relevant, as discussed below^35, 47, 48^.

The short strands in E2 HVR2 and IgVR form a three-stranded, antiparallel β-sheet that was not previously resolved in E2c structures, either because they were not present in the construct or, likely, misfolded due to the absence of E1. The C-terminal region, not included in E2c, is characterized by the presence of two short β-strands and one extended α-helix (residues 684–697); intriguingly, the region spanning residues 671–705 has been recently shown to be involved in the pH-induced conformational changes required for fusion through the interaction with E1, as both the peptide itself and a mAb against it abrogate infection^49^. The identified secondary structure elements, and their arrangement in E1E2 quaternary structure, may help in the rational design of fusion inhibitors.

Contrary to E2, whose structure prediction was aided by E2c crystals, E1 structure was determined *ab initio*. In fact, although a structure for the E1 N-terminal domain was recently published, it was not biologically or functionally validated and assembled in disulfide-bound homodimers, while E1 native state on virions is a non-covalent homotrimer^6, 9^. Furthermore, our cysteine coupling analysis could not confirm any of the disulfide bridges highlighted in the crystal structure, suggesting a non-native fold. These aspects may be due to the usage of a construct that excluded four cysteines and, as highlighted by our prediction, several β-strands, that most likely have altered E1 N-terminal overall conformation, disulfide connectivity and oligomerization state.

The E1 topology we have predicted presents two four-stranded, antiparallel β-sheets, which are positioned parallel to each other, and a central, bipartite, α-helix spanning residues 256–266 and 269–291. The helical structures in E1 have been intensively studied using HCVcc and HCVpp mutants, and led to the identification of several residues involved in virus fusion and heterodimerization. Specifically, the extended helical region spanning residues 256–288 appears to possess a dual role; all tested mutations abrogate fusion, some of them directly and some others through the abrogation of E1E2 heterodimerization^50, 51^. The role of this region in fusion is confirmed by HCVcc escape mutations to Flunarizine and a 4-aminoquinoline derivative (B5), two fusion inhibitors, that occurred at residues 267 and 289 for the former and at 291 for the latter^12, 14^. Intriguingly, we have identified an inter-monomer disulfide bond involving E1 C272 comprised in the central helix; when either C272 or its partner on E2, C452, is mutated, the binding of all tested mAbs is abrogated, suggesting E1E2 misfolding. Therefore, the C272-C452 inter-monomer disulfide bond appears to be essential for heterodimerization and correct folding, strengthening the evidence by Falson *et al.* and Wahid *et al.* of the sequential assembly of E1 and E2 in disulfide-linked heterodimers that subsequently trimerize to form the functional, heterohexameric HCV entry machinery^9, 52^. Moreover, although not supported by direct biological data, it can be speculated that the E1 central helix plays a role in the formation of the E1 homotrimer.

The intermonomer disulfide bridge is only one of the E1-E2 ectodomain interfaces participating in the native E1E2 conformation; our model describes in fact the structural features of two other heterodimerization interfaces. While the interface formed by residues 299–303 and 671–675 is proposed here for the first time, several pieces of evidence supporting the interaction between E1 residues 219–223 and E2 569–573 are available in literature. McCaffrey *et al.* first demonstrated that both HVR2 and IgVR are required for heterodimerization; subsequently, Maurin *et al*. identified E1 MIM/AIL domain (residues 219–221) as a major determinant for E1-E2 intergenotypic compatibility^34, 53^. Overall, our model provides a structural explanation to the fundamental role played by these regions in heterodimerization; furthermore, it suggests the erroneous folding path that lead to E2c cysteine mispairing. The expression of E2 in the absence of E1 causes C452 to be without a partner; as a consequence, in 4MWF it couples with C620, which is sequestered from its native partner, C459. Moreover, in the absence of E1, HVR2 and IgVR correct folding, as well as the formation of the native C486-C581 and C569-C585 disulfide bridges, cannot be achieved, as highlighted by the capability of H60 to bind E2 only upon heterodimerization and when all four cysteines are present.

The human humoral immune response mainly targets E2, while E1 seems to be less immunogenic, with the majority of anti-E1 mAbs recognizing linear epitopes; moreover, only few neutralizing mAbs have been isolated to date^54, 55^. The difference in immunogenicity led to the hypothesis of E2 being more exposed, while E1 is shielded by both E2 and the apolipoproteins captured during budding^56, 57^. In our model, E1 locates distally considering the heterodimer conformation and is therefore compatible with it being poorly accessible on virions. The direct interaction of HVR2 and IgVR with E1, together with HVR1 localization, suggests a novel role for E2 hypervariable regions in shielding both E2 itself and E1. The exposure of E1 and E2 to the immune system is further limited by the presence of 16 N-glycosylation sites (five in E1 and eleven in E2) and five O-glycosylation sites that were represented in our model. Besides assuring the correct exposure and local folding of regions associated with the glycan moieties, the structure-function relationship of E1E2 glycosylations clearly emerges from the model. Glycans bound to residues N417, N423, N430, T518, N532 and N556 directly protect the CD81bs, while N448 and O-glycosylations in HVR1 (T385, T396, T404) have a dual role in protecting the CD81bs and E1 apical regions. Glycosylations in HVR2 and IgVR (S473, N476, N576), as well as E2 glycosylations at N623 and N645 and E1 N196, N209, N234 and N305 mainly cover the lateral regions of E1 and E2. Finally, E2 N540 shields the Ig-like domain upper sheet (Figure S6). Overall, our model provides a structural insight into E1 and E2 immunogenicity and HCV immune evasion.

The E1E2 complex requires several sequential steps to achieve fusion. Upon attachment, which is mainly mediated by virion-associated apoE, E2 HVR1 interacts with SR-BI, causing a local rearrangement that exposes the CD81bs^58^. The subsequent interaction between E2 and CD81 causes a first conformational change that involves the rearrangement of E1E2 disulfide bridges mediated by free cysteines, as demonstrated by the irreversible inhibitory effect of free cysteines alkylation^24^. Following disulfide isomerization, E1 and E2 require a second trigger, pH lowering, to undergo the conformational changes that lead to fusion. Our model allows each domain in the E1E2 heterodimer structure to be correlated with function; furthermore, its native, pre-attachment disulfide connectivity, which was determined using conformational mAbs, provides information on the disulfide isomerization process and the involved cysteines.

**Figure 6:**
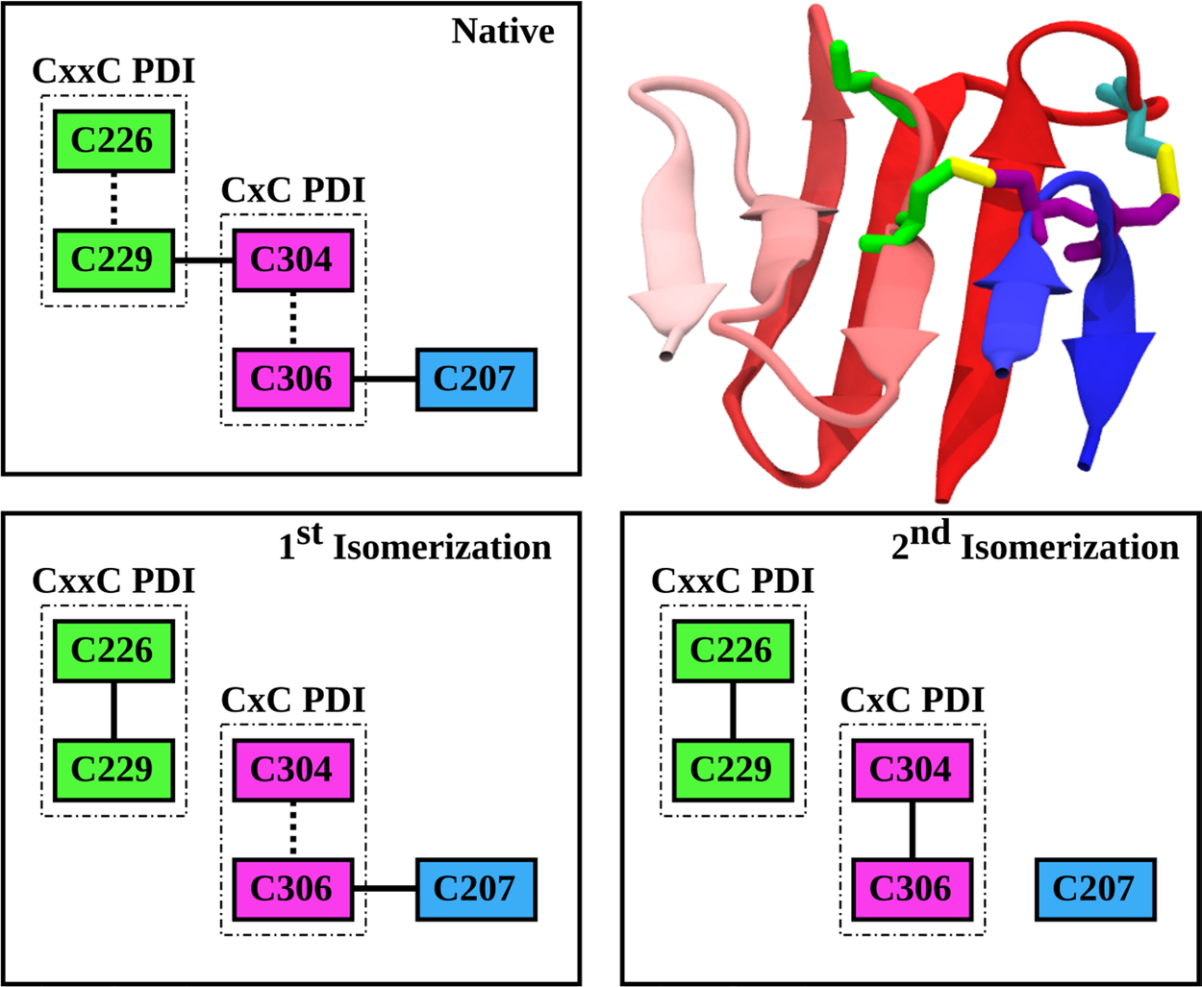
E1 disulfide isomerization. E1 β-sheets structure in the native conformation is depicted in cartoon and colored according to residue position (red residues are located at the N-terminal, blue residues at the C-terminal) to highlight long-range β-strand coupling. Cysteines are represented in licorice and colored according to the scheme. In the three boxes, cysteine coupling in native conditions and after the first and second disulfide isomerization steps are represented. Continuous lines connecting each cysteine represent disulfide bridges, while dashed lines highlight proximal cysteines in the linear sequence.

The first direct interaction between HCV and host receptors involves E2 HVR1 and host SR-BI^56, 59^. In our model the short 385–388 β-strand couples parallel to epitope I β-hairpin; it can be speculated that, considering the weak nature of parallel β-sheet hydrogen bonds, this coupling could be broken upon HVR1-SR-BI interaction, causing CD81bs exposure. This hypothesis is strengthened by several pieces of evidence suggesting that neutralizing conformational antibodies targeting epitope I β-hairpin bind at a post-attachment step; considering the association of the 385–388 strand to the β-hairpin, this epitope may become available only after SR-BI strand displacement^60, 61^. Following HVR1 repositioning, E2 interacts with CD81, triggering a rearrangement in disulfide connectivity mediated by free cysteines. Wahid *et al.* have speculated that E1 C226 and C229 form a classic CxxC protein disulfide isomerization (PDI) domain analogous to that of Env fusion proteins from β-, γ-, δ-retroviruses^52, 62, 63^. It should also be noted that Woycechowsky *et al*. have characterized a CxC PDI sequence, also present in E1 at positions 304–306^64^. In our model, C226 is in a free state in the pre-attachment heterodimer, as expected for a PDI domain in which it is always the N-terminal cysteine to carry on the first nucleophilic attack, and can therefore induce disulfide isomerization during fusion; the formation of a C226-C229 bond would cause the breakage of the C229-C304 long-range bond. Furthermore, if C304 and C306 also constitute a PDI, C304 would become the active-site thiolate upon the first isomerization, resulting in the formation of two new, short-range disulfide bridges, C226-C229 and C304-C306 (Fig. 6), which in turn would induce the loss of E1 globular conformation and uncover the putative central E1 fusion domain. In conclusion, our model enables speculations regarding the molecular processes underlying the conformational changes required for the sequential receptors recognition and virus fusion.

Our model is based on both computational analyses and, more importantly, biological data derived from alanine scanning performed on the entire HCV E1E2 sequence. The reactivity pattern of a panel of human antibodies against the native structure was fundamental to obtain useful information in the modeling phase and to validate the model itself. Although E2c crystals unveiled for the first time the structure of conformational epitopes overlapping the CD81bs, no structural information has been derived for other epitopes. Here we describe for the first time the structural features of other rare epitopes for both neutralizing and non-neutralizing antibodies, helping to understand the immune response against E1 and E2 in their native conformation.

To conclude, the E1E2 heterodimer model we have generated represents a step towards the fine characterization of HCV entry machinery, filling the gaps left by truncated E1 and E2 crystal structures. Information derived from the model could influence both further structural studies and the development of novel prophylactic and therapeutic strategies. The model could in fact be used to rationally generate truncated E1 and E2 constructs for structural studies without incurring in the introduction of biases that burden current crystals. Moreover, an accurate knowledge of E1E2 heterodimer structure represents a step toward the definition of E1E2 heterohexamer, which still requires a reliable structure characterization. Finally, the structural model described here could also provide a starting point for targeted drug discovery and the generation of immunogens.

## Materials and Methods

### Construction of HCV E1E2 mutant library

A library of 553 single-point alanine mutants was created for HCV (strain H77, Uniprot accession number F5BWY6) envelope proteins E1 and E2 (residues 192–746 of the HCV polyprotein, henceforth used as the reference numbering) through shotgun mutagenesis, as reported elsewhere^25, 65^. Alanine residues in the wild-type sequence were mutated into serine. A C-terminal V5-His tag was included to assess construct expression level. Each individual mutation was verified by DNA sequencing.

### Mabs

Human mAbs e8, e10, e20, e137, e301 and e509 were isolated as Fab fragments from a phage display antibody library produced from bone marrow lymphocytes of a chronically-infected donor as previously described^17^. Fabs were produced in *E. coli*. The detailed expression and purification protocols are described elsewhere^66^. Mab H60 (IgG1 form) is a generous gift from Professor J. Debuisson.

### Immunofluorescence assay

HEK-293T cells were plated in 384-well microplates and transfected with each E1E2 mutant construct (one mutant per well). Transfected cells were fixed in 4% paraformaldehyde in phosphate-buffered saline (PBS). The cells were incubated with anti-E2 antibody for 1 h at room temperature, followed by a 30-min incubation with Alexa Fluor 488-conjugated secondary antibody (Jackson ImmunoResearch Laboratories, West Grove, PA) in 10% normal goat serum (NGS). Cells were washed twice with PBS without calcium or magnesium (PBS−/−) and resuspended in Cellstripper (Cellgro, Manassas, VA) plus 0.1% bovine serum albumin (BSA; Sigma-Aldrich, St. Louis, MO). Cellular fluorescence was detected using an Intellicyt high-throughput flow cytometer (Intellicyt, Albuquerque, NM). Background fluorescence was determined by fluorescence measurement of vector-transfected control cells. MAb reactivities against each E1E2 mutant clone were calculated relative to wild-type reactivity after subtracting the signal from mock-transfected controls^25, 65^.

### E1E2 sequence collection

E1E2 full length sequences (residues 192–746) were retrieved from the Hepatitis C Virus Database Project^67^. A first multiple sequence alignment (MSA) comprising 5347 sequences was generated using Clustal Omega and subsequently filtered to remove identical or truncated sequences. The refined MSA used for further analysis comprised 2971 E1E2 FL sequences^68, 69^.

### Secondary structure prediction

HCV E1E2 secondary structure was predicted using the best performing secondary structure algorithms. Algorithms exploiting MSAs were fed with the refined E1E2 MSA, while single sequence-based algorithms received the H77 E1E2 reference sequence. Specifically, JPRED4, PSIPRED and NetSurfP received E1E2 MSA, while s2D, DeepConCNF, Sable and PSSpred received H77 E1E2 single sequence^70–75^. All the prediction algorithms were used with the default parameters. The final secondary structure used for further analyses was determined using a majority voting approach on the output of each algorithm.

### Evolutionary Coupling Analysis

Six EC algorithms (metaPSICOV, ccmPRED, PConsC2, plmDCA, EPC-MAP and RaptorX) were selected to predict all possible E1E2 residue couples’ propensity of interaction^38, 76–80^. When possible, the algorithm was fed with the refined E1E2 alignment (plmDCA), while metaPSICOV, PConsC2, EPC-MAP, ccmPRED and RaptorX web servers received the single H77 E1E2 sequence. In order to select the algorithm that reached the best performance for E1E2, predicted contact maps were compared to the real contact map of the E2 Ig-like domain (PDB ID: 4MWF, residues 494–566). The contact map was calculated by considering two residues in contact when their carbon alpha (Cα) were closer than 10 Å. Predicted contact maps were trimmed to include only the residues of the 4MWF Ig-like domain. Subsequently, predicted couples were ordered from highest to lowest score and compared with the real contact map. At each iteration, the positive predictive value (PPV) was calculated.

### Beta sheets pairing prediction

The bbcontacts algorithm was used to predict the β-strand pairing in E1E2^39^. The algorithm was applied to the refined secondary structure prediction and the contact map defined by RaptorX. The predicted contact map was manually modified to include real contacts in the Ig-like domain and E2 C-terminal β-sheet (residues 602–645) derived from 4MWF crystal structure. As described before, the distance threshold used to generate 4MWF contact map was a Cα-Cα distance below 10 Å.

### Dimer model generation and validation

E1 and E2 ectodomains (residues 192–327 and 384–711) were modeled in their heterodimeric form using the CNS suite^40, 41^. Secondary structure distance restraints for α-helices and β-strands were taken from Marks *et al.* ^37^. H-bonds defining β-sheets strand pairing were taken from the E2c crystal structures for resolved domains, while for predicted domains they were defined forcing two standard distances, N-O and H-O, respectively of 2.8 Å and 1.8 Å. Finally, tertiary structure distance restraints were either measured from E2c structures or assigned on the basis of RaptorX predicted couples. In both cases, only couples spaced by at least 24 residues were taken into account. Moreover, EC residue couples involving residues belonging to the same domain (i.e. lying in the same β-sheet) were excluded. All the restraints and the unfolded structures of E1 and E2 carrying glycosylations and disulfide bridges were used to predict the E1E2 heterodimer structure with a distance geometry simulated annealing (DG/SA) protocol. In details, DG was applied to generate a first embedded structure satisfying the restraints that was subsequently refined by SA simulation. The DG/SA procedure followed the standard CNS protocol with an extended number of steps for SA slow-cool annealing dynamics (25000). A hundred structures were generated and clustered with VMD on the basis of backbone atoms RMSD. The final E1E2 heterodimer model was selected as the centroid of the most populated cluster; an unrestrained minimization of 10000 steps was subsequently performed with CNS. Epitope residues were mapped on the final model to evaluate the correct clustering and exposure using VMD 1.9.2^81^.

## Electronic supplementary material

Supplementary Information
