# Supplementary material for: A Biologically-validated HCV E1E2 Heterodimer Structural Model
Source: Sci Rep. 2017 Mar 16;7:214. doi: 10.1038/s41598-017-00320-7 (PMC5428263; doi:10.1038/s41598-017-00320-7)
Supplement: Supplementary file 1 — Supplementary Information [file 41598_2017_320_MOESM1_ESM.pdf]

A Biologically-validated HCV E1E2 Heterodimer Structural Model

Matteo Castelli[1], Nicola Clementi[1], Jennifer Pfaff[2], Giuseppe A. Sautto[1], Roberta A. Diotti[1], Roberto Burioni[1], Benjamin J. Doranz[2], Matteo Dal Peraro[3,4], Massimo Clementi[1], Nicasio Mancini[1*]

[1]Laboratory of Microbiology and Virology, Università "Vita-Salute" San Raffaele, Via Olgettina 58, 20132 Milano, Italy
[2]Integral Molecular, 3711 Market St #900, Philadelphia, PA 19104, USA
[3]Laboratory for Biomolecular Modeling, Institute of Bioengineering, School of Life Sciences, Ecole Polytechnique Fédérale de Lausanne, Route Cantonale, 1015 Lausanne, Switzerland
[4]Swiss Institute of Bioinformatics, Lausanne, Switzerland

E1

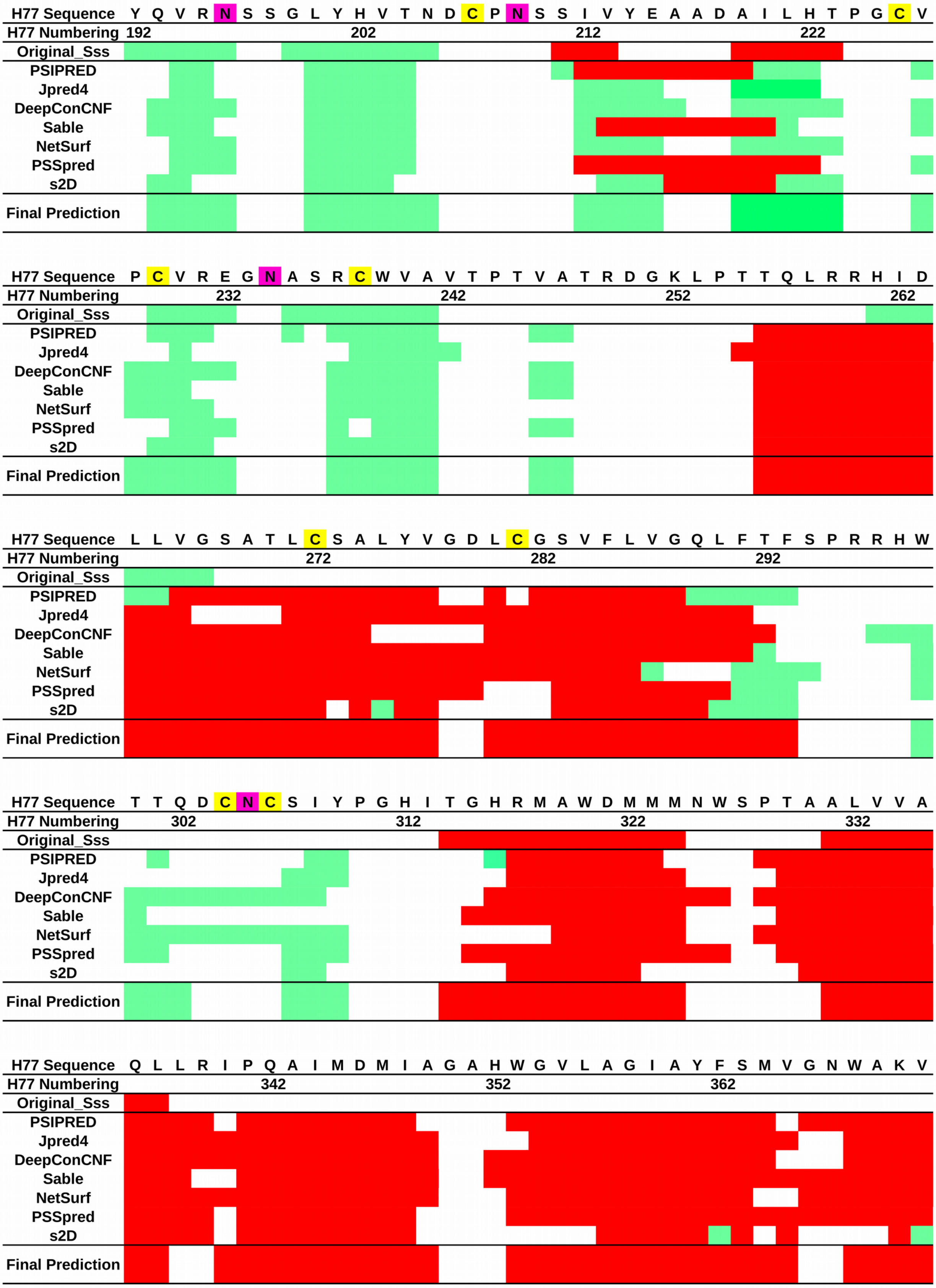
H77 Sequence Y Q V R N S S G L Y H V T N D C P N S S I V Y E A A D A I L H T P G C V
H77 Numbering 192 202 212 222
Original_Sss
PSIPRED
Jpred4
DeepConCNF
Sable
NetSurf
PSSpred
s2D
Final Prediction
H77 Sequence P C V R E G N A S R C W V A V T P T V A T R D G K L P T T Q L R R H I D
H77 Numbering 232 242 252 262
Original_Sss
PSIPRED
Jpred4
DeepConCNF
Sable
NetSurf
PSSpred
s2D
Final Prediction
H77 Sequence L L V G S A T L C S A L Y V G D L C G S V F L V G Q L F T F S P R R H W
H77 Numbering 272 282 292
Original_Sss
PSIPRED
Jpred4
DeepConCNF
Sable
NetSurf
PSSpred
s2D
Final Prediction
H77 Sequence T T Q D C N C S I Y P G H I T G H R M A W D M M M N W S P T A A L V V A
H77 Numbering 302 312 322 332
Original_Sss
PSIPRED
Jpred4
DeepConCNF
Sable
NetSurf
PSSpred
s2D
Final Prediction
H77 Sequence Q L L R I P Q A I M D M I A G A H W G V L A G I A Y F S M V G N W A K V
H77 Numbering 342 352 362
Original_Sss
PSIPRED
Jpred4
DeepConCNF
Sable
NetSurf
PSSpred
s2D
Final Prediction

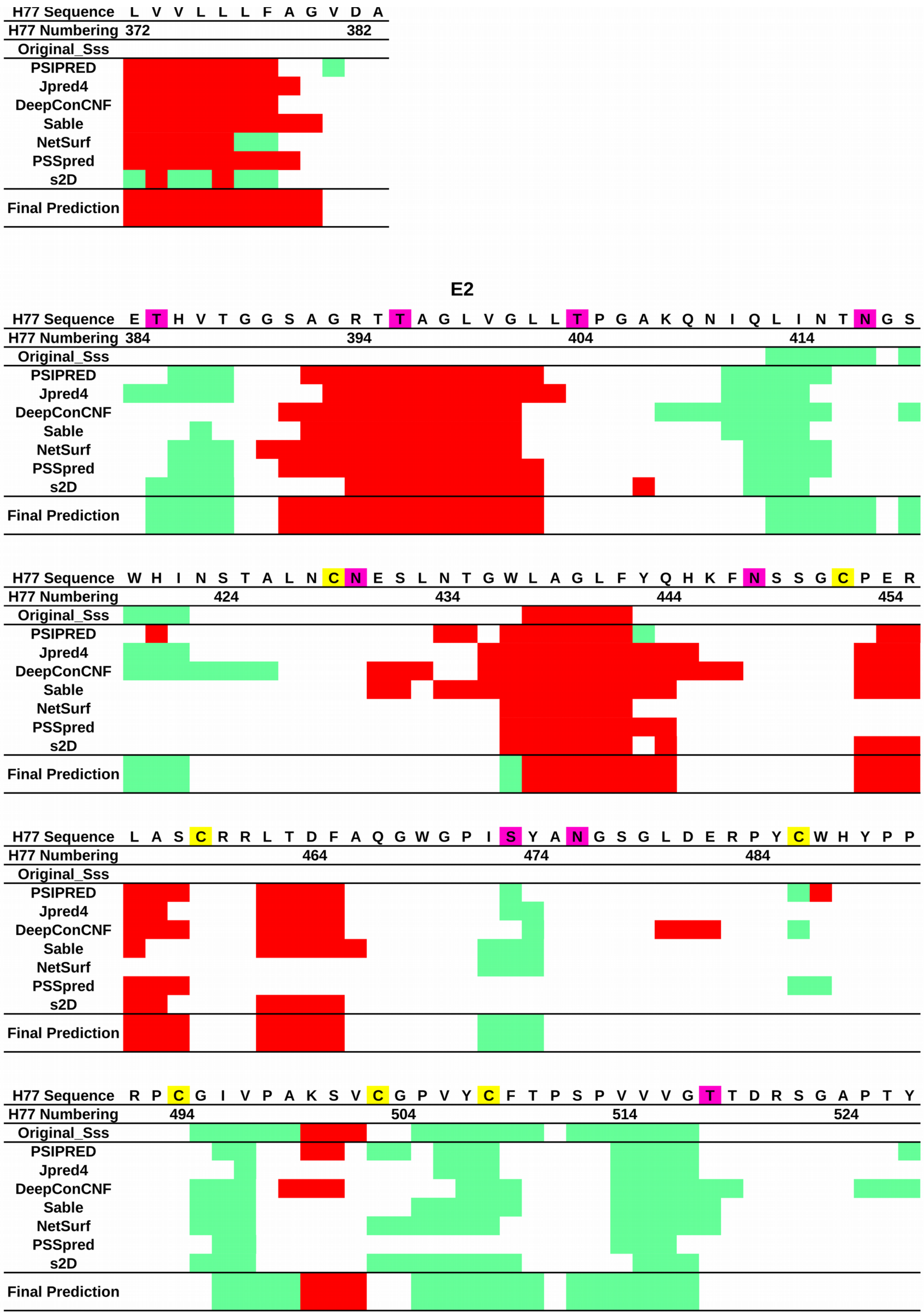
H77 Sequence L V V L L L F A G V D A
H77 Numbering 372 382
Original_Sss
PSIPRED
Jpred4
DeepConCNF
Sable
NetSurf
PSSpred
s2D
Final Prediction
E2
H77 Sequence E T H V T G G S A G R T T A G L V G L L T P G A K Q N I Q L I N T N G S
H77 Numbering 384 394 404 414
Original_Sss
PSIPRED
Jpred4
DeepConCNF
Sable
NetSurf
PSSpred
s2D
Final Prediction
H77 Sequence W H I N S T A L N C N E S L N T G W L A G L F Y Q H K F N S S G C P E R
H77 Numbering 424 434 444 454
Original_Sss
PSIPRED
Jpred4
DeepConCNF
Sable
NetSurf
PSSpred
s2D
Final Prediction
H77 Sequence L A S C R R L T D F A Q G W G P I S Y A N G S G L D E R P Y C W H Y P P
H77 Numbering 464 474 484
Original_Sss
PSIPRED
Jpred4
DeepConCNF
Sable
NetSurf
PSSpred
s2D
Final Prediction
H77 Sequence R P C G I V P A K S V C G P V Y C F T P S P V V V G T T D R S G A P T Y
H77 Numbering 494 504 514 524
Original_Sss
PSIPRED
Jpred4
DeepConCNF
Sable
NetSurf
PSSpred
s2D
Final Prediction

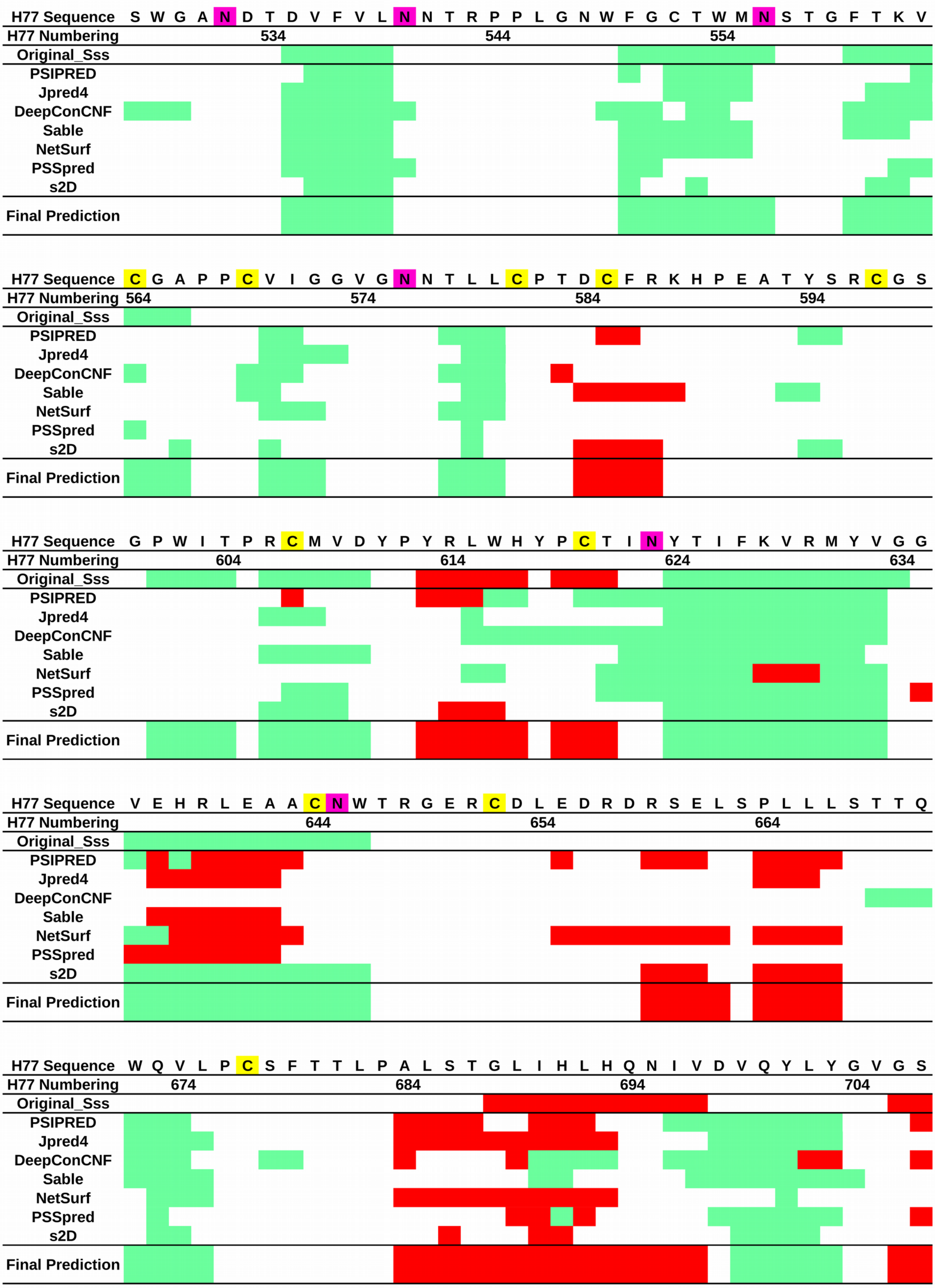
H77 Sequence S W G A N D T D V F V L N N T R P P L G N W F G C T W M N S T G F T K V
H77 Numbering 534 544 554
H77 Sequence C G A P P C V I G G V G N N T L L C P T D C F R K H P E A T Y S R C G S
H77 Numbering 564 574 584 594
H77 Sequence G P W I T P R C M V D Y P Y R L W H Y P C T I N Y T I F K V R M Y V G G
H77 Numbering 604 614 624 634
H77 Sequence V E H R L E A A C N W T R G E R C D L E D R D R S E L S P L L L S T T Q
H77 Numbering 644 654 664
H77 Sequence W Q V L P C S F T T L P A L S T G L I H L H Q N I V D V Q Y L Y G V G S
H77 Numbering 674 684 694 704
Original_Sss
PSIPRED
Jpred4
DeepConCNF
Sable
NetSurf
PSSpred
s2D
Final Prediction

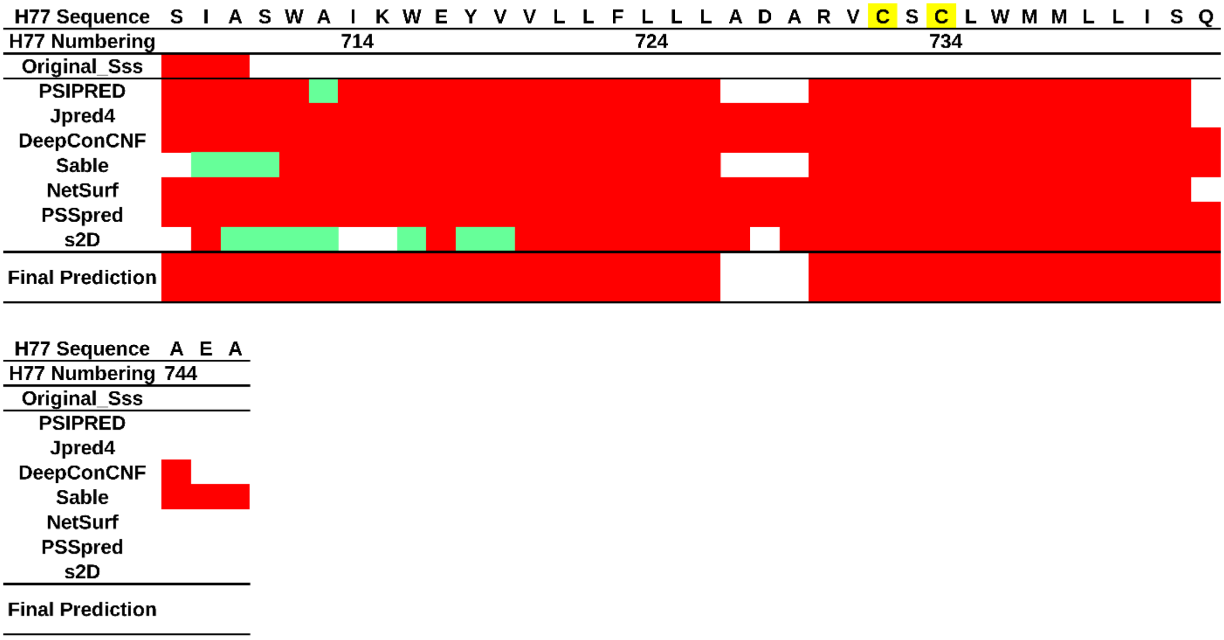

Figure S1: ***FL E1E2 secondary structure prediction*.** The output of each secondary structure prediction algorithm is reported, with α-helices in red and β-strands in green. The final prediction used for the subsequent steps and the secondary structures derived from crystallographic and NMR structures are reported as well. The reference sequence reports cysteines and glycosylation sites in yellow and purple, respectively.

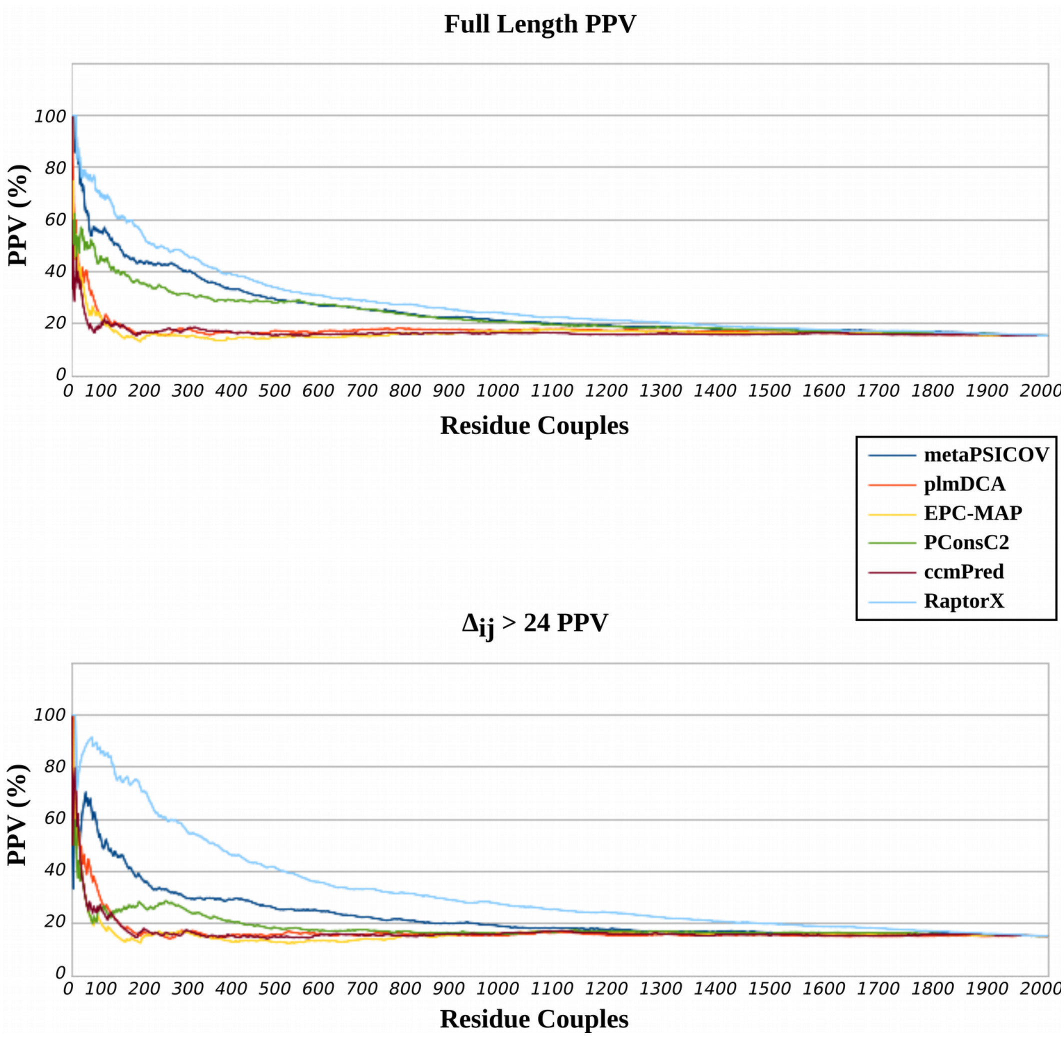

Figure S2: ***EC algorithms comparison.*** The performance of each tested evolutionary coupling algorithm is reported as the PPV calculated on E2 Ig-like domain contact map at 10Å. On the X-axis is presented the number of residue couples compared, with decreasing score; on y-axis is reported the PPV as a percentage.

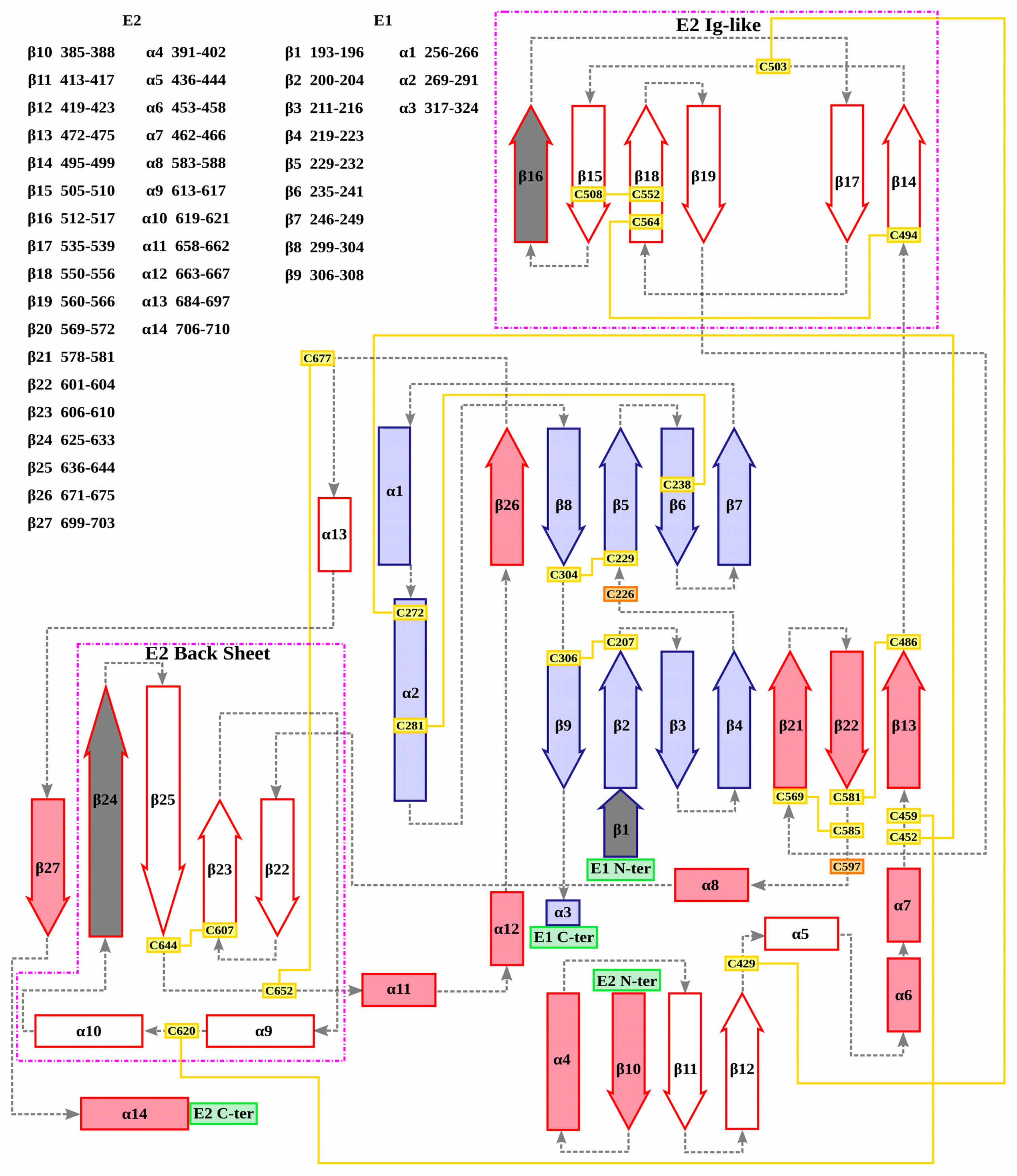

Figure S3: ***E1E2 topology*****.** The entire proteins predicted topology is reported with α-helices as rectangles and β-strands as arrows. Blue and red outlines indicate E1 and E2 secondary structures, respectively. Newly identified secondary structures are represented in blue and red fill (for E1 and E2 elements, respectively), white fill indicates the correspondence between the predicted secondary and tertiary structures and those present in E2c, gray fill indicates a mismatch between predictions and E2c structures. Successive secondary structure elements are connected by dashed gray lines.

Cysteines involved in a disulfide bridge are boxed in yellow and connected by yellow lines; cysteines boxed in orange are free.

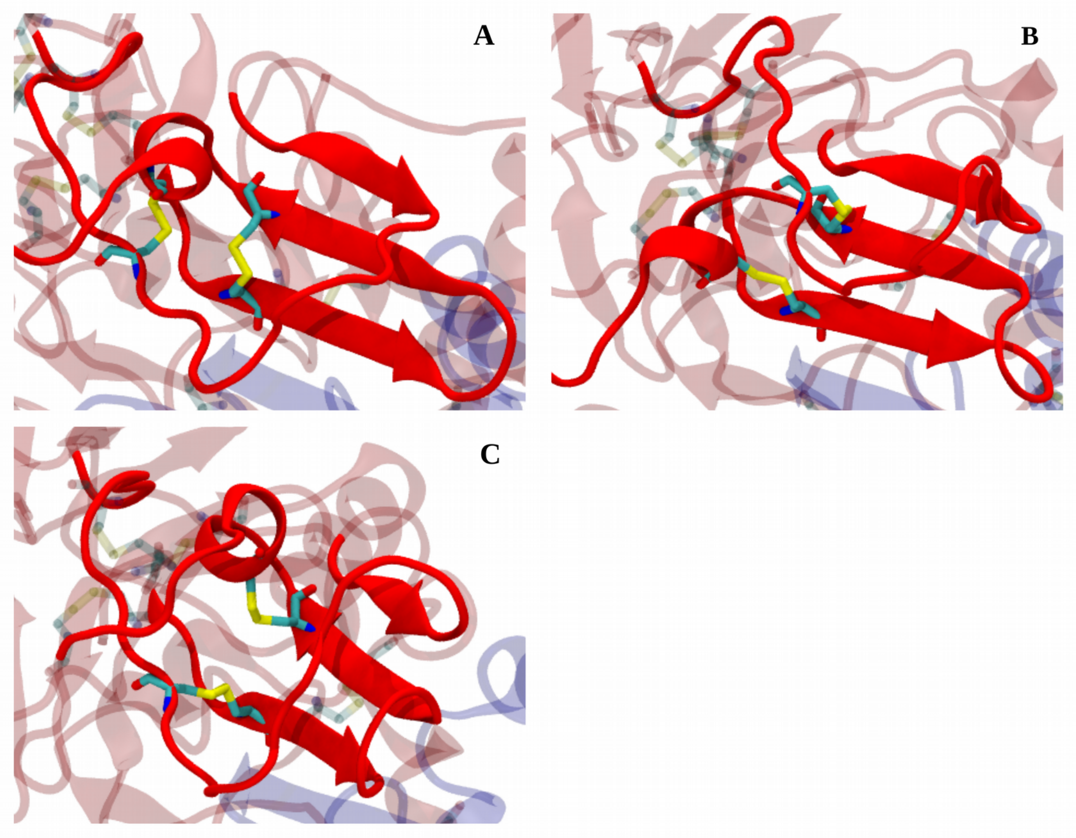

Figure S4: ***Disulfide connectivity involving cysteines C486, C569, C581 and C585.*** The three different arrangements involving HVR2 and IgVR cysteines are depicted with variable regions in solid red, while E1 and other E2 domains are in transparent blue and red, respectively. The systems carried the subsequent disulfide bridges: panel **A** C486-C585 and C569-C581, panel **B** C486-C581 and C569-C585 and panel **C** C486-569 and C581-C585.

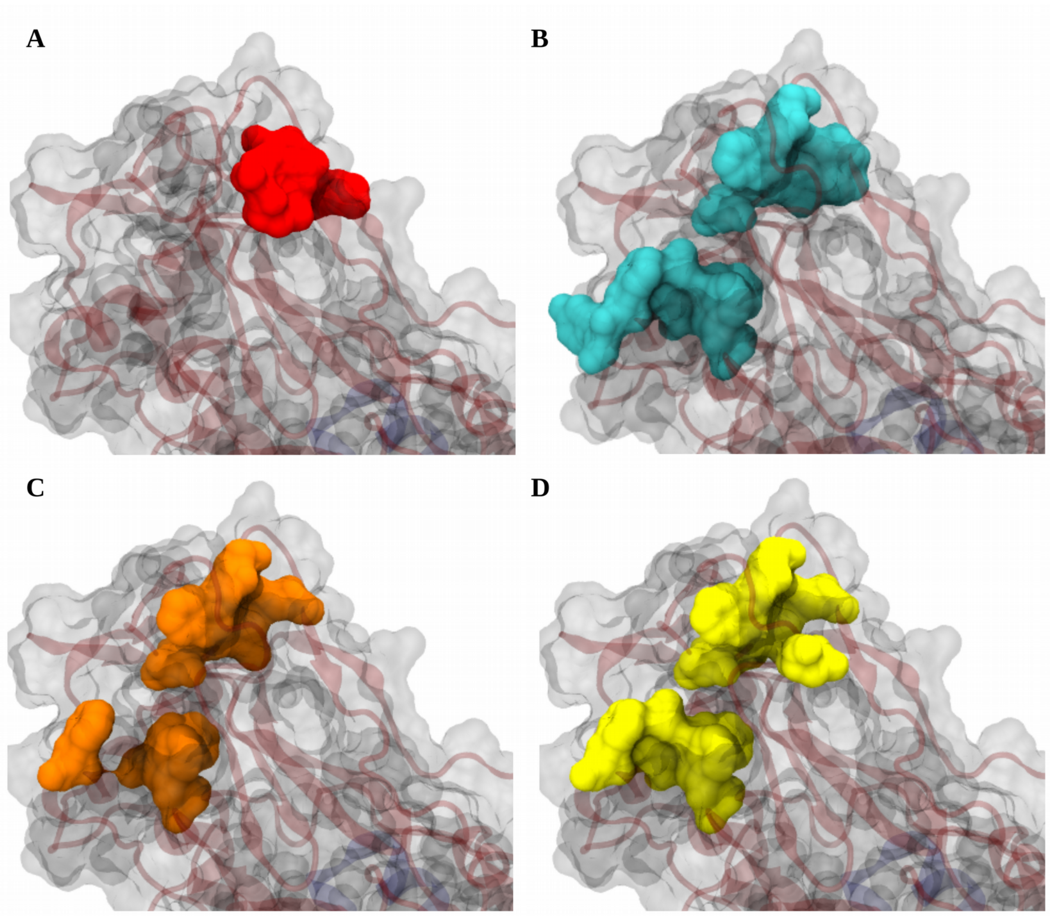

Figure S5: ***Epitope mapping of mAbs e10, e20, e301 and e509.*** Residues involved in binding to a non-neutralizing antibody (e10, panel **A**), a weak neutralizer (e509, panel **B**), and two broadly neutralizing antibodies (e20 in panel **C** and e301 in panel **D**) are mapped on E1E2 model.

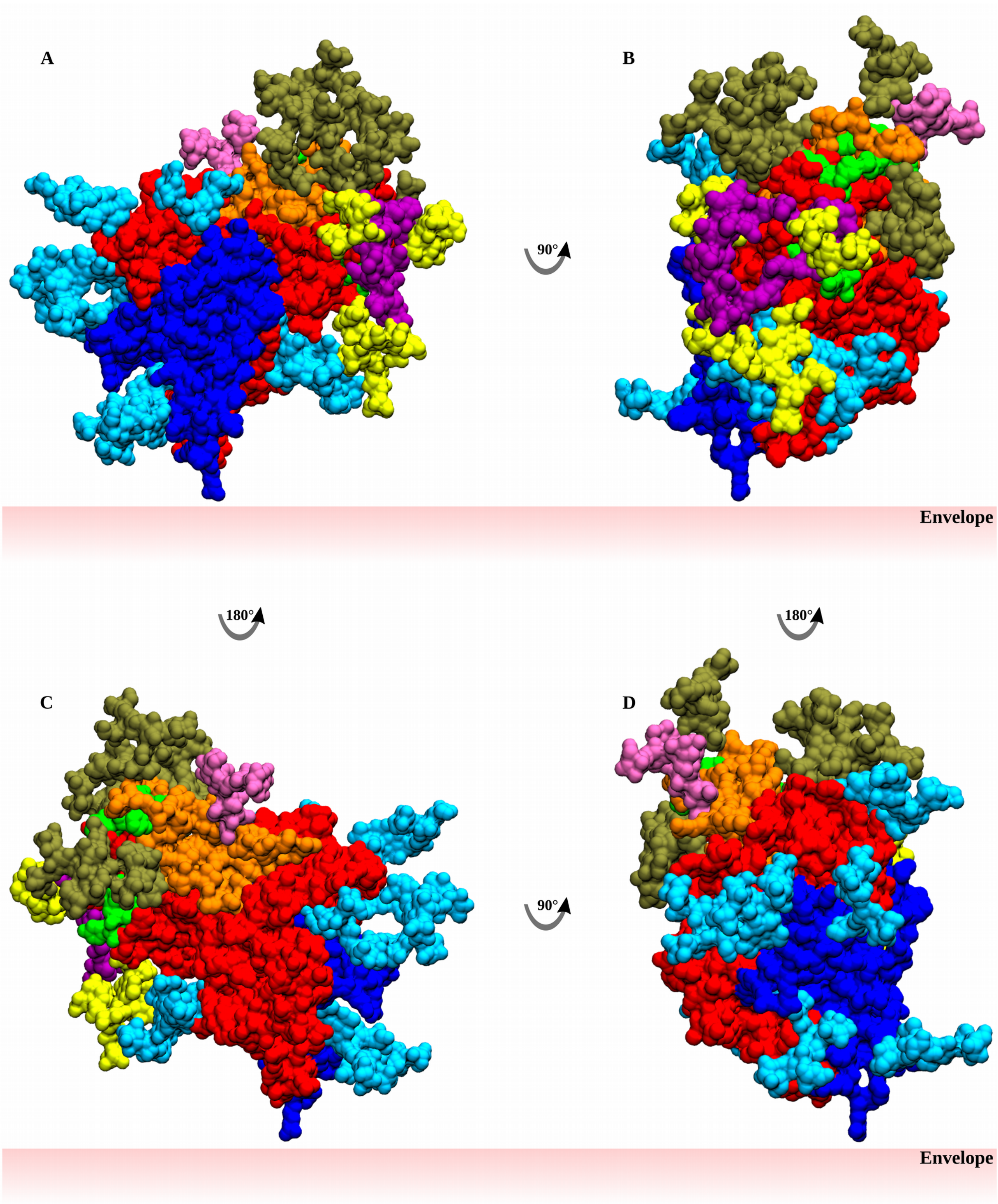

Figure S6: ***E1E2 glycosylations*****.** The model is depicted in surface representation, with glycan moieties colored differently according to the protein region they shield. Consistently with Figure 4 in the main text, E1, E2, the Ig-like domain, HVR1 and CD81bs are represented with the same coloring scheme (respectively blue, red, orange, purple and green). Glycosylations are grouped and colored as follows: those protecting exclusively the CD81bs are in tan, those shielding the CD81bs

and E1 are in yellow, those covering the lateral domains of E1 and E2 are in cyan and N540, that protects the Ig-like domain upper layer, in pink. Panel **A** and **C** represent lateral views, panel **B** a front view and panel **D** a back view of the model.
